# Analysis of CXCL9, PD1 and PD-L1 mRNA in Stage T1 Non-Muscle Invasive Bladder Cancer and Their Association with Prognosis

**DOI:** 10.3390/cancers12102794

**Published:** 2020-09-29

**Authors:** Jennifer Kubon, Danijel Sikic, Markus Eckstein, Veronika Weyerer, Robert Stöhr, Angela Neumann, Bastian Keck, Bernd Wullich, Arndt Hartmann, Ralph M. Wirtz, Helge Taubert, Sven Wach

**Affiliations:** 1 Department of Urology and Pediatric Urology, University Hospital Erlangen, Friedrich-Alexander Universität Erlangen-Nürnberg, 91054 Erlangen, Germany; jennifer.kubon@fau.de (J.K.); danijel.sikic@uk-erlangen.de (D.S.); Angela.Neumann@uk-erlangen.de (A.N.); bastian.keck@web.de (B.K.); Bernd.Wullich@uk-erlangen.de (B.W.); sven.wach@uk-erlangen.de (S.W.); 2 Institute of Pathology, University Hospital Erlangen, Friedrich-Alexander Universität Erlangen-Nürnberg, 91054 Erlangen, Germany; Markus.Eckstein@uk-erlangen.de (M.E.); veronika.weyerer@uk-erlangen.de (V.W.); robert.stoehr@uk-erlangen.de (R.S.); arndt.hartmann@uk-erlangen.de (A.H.); 3 STRATIFYER Molecular Pathology GmbH, 50935 Cologne, Germany; ralph.wirtz@stratifyer.de

**Keywords:** *CXCL9*, *PD1*, *PD-L1*, stage T1 NMIBC, prognosis

## Abstract

**Simple Summary:**

Non-muscle invasive bladder cancer (NMIBC) patients possess a high rate of recurrences and very long treatment times, which remains a major unresolved problem for them and the health care system. We analyzed the mRNA of three immune markers, *CXCL9, PD1* and *PD-L1*, in 80 NMIBC by qRT-PCR. Lower *CXCL9* mRNA appeared to be an independent prognostic parameter for reduced OS and RFS. Furthermore, low *PD-L1* mRNA was an independent prognostic factor for DSS and RFS. In univariate Cox’s regression analysis, the stratification of patients revealed that low *CXCL9* or *PD1* mRNA was associated with reduced RFS in the patient group younger than 72 years. Low *CXCL9* or *PD-L1* was associated with shorter RFS in patients with higher tumor cell proliferation or without instillation therapy. In conclusion, the characterization of mRNA levels of the immune markers *CXCL9*, *PD1* and *PD-L1* differentiates NIMBC patients with respect to prognosis.

**Abstract:**

Non-muscle invasive bladder cancer (NMIBC), which is characterized by a recurrence rate of approximately 30% and very long treatment times, remains a major unresolved problem for patients and the health care system. The immunological interplay between tumor cells and the immune environment is important for tumor development. Therefore, we analyzed the mRNA of three immune markers, *CXCL9*, *PD1* and *PD-L1*, in NMIBC by qRT-PCR. The results were subsequently correlated with clinicopathological parameters and prognostic data. Altogether, as expected, higher age was an independent prognostic factor for overall survival (OS) and disease-specific survival (DSS), but not for recurrence-free survival (RFS). Lower *CXCL9* mRNA was observed in multivariate Cox’s regression analysis to be an independent prognostic parameter for reduced OS (relative risk; RR = 2.08; *p* = 0.049), DSS (RR = 4.49; *p* = 0.006) and RFS (RR = 2.69; *p* = 0.005). In addition, *PD-L1* mRNA was an independent prognostic factor for DSS (RR = 5.02; *p* = 0.042) and RFS (RR = 2.07; *p* = 0.044). Moreover, in univariate Cox’s regression analysis, the stratification of patients revealed that low *CXCL9* or low PD1 mRNA was associated with reduced RFS in the younger patient group (≤71 years), but not in the older patient group (>71 years). In addition, low *CXCL9* or low *PD-L1* was associated with shorter RFS in patients with higher tumor cell proliferation and in patients without instillation therapy. In conclusion, the characterization of mRNA levels of immune markers differentiates NIMBC patients with respect to prognosis.

## 1. Introduction

Urothelial bladder cancer (BCa) accounts for approximately 3% of global cancer diagnoses. It was recently reported to be the 10th most commonly diagnosed cancer and the 13th leading cause of cancer-related death worldwide [1]. Approximately 25% of BCas are categorized as muscle-invasive BCa (MIBC) and 75% as non-muscle invasive BCa (NMIBC) [2]. NMIBC treatment comprises transurethral resection of the bladder (TURB) and, depending on the risk of progression, instillation with bacillus Calmette-Guerin (BCG) or mitomycin [3,4,5]. However, high-risk NMIBC remains a challenge because 30% to 60% of patients with stage pT1 NMIBC develop local recurrence, and up to 20% experience disease progression to MIBC [6,7,8]. There is heterogeneity in stage pT1 NMIBC, and its risk stratification is based only on clinicopathological parameters that necessitate lifelong follow-up [9]. Altogether, bladder cancers, including NMIBC, impose the highest costs on society among cancers per patient from diagnosis to death [10]. However, bladder tumor markers cannot yet definitively replace cystoscopy in surveillance regimens [10]. Therefore, the continued search for biomarkers in bladder cancer is necessary.

The tumor biology of BCa, including NMIBC, is related to cell lineage and cell proliferation [11,12,13]. Therefore, we included an analysis of the mRNA of keratin 5 (*KRT5*; basal-like lineage), keratin 20 (*KRT20*; luminal-like lineage) and marker of proliferation KI67 (*MKI67*, *KI67*) in this study. Furthermore, studies conducted by other groups, as well as our own previous studies, showed that gene expression can differentiate NMIBCs into subsets that possess different risk profiles, and may impact treatment decisions in the future [14,15].

In the current study, we investigated the expression of genes associated with tumor immune status and their association with prognosis in stage pT1 NMIBC. Recently, we reported that a cytotoxic T-cell-related gene expression signature containing three genes (*CXCL9*, *CD3 Z*, *CD8*) correlates with immune cell infiltration, and predicts improved survival in MIBC patients after radical cystectomy and adjuvant chemotherapy [16]. All three immune signature genes were strongly associated with each other, which is why we chose only *CXCL9* for the current analysis. Additionally, we chose programmed cell death 1 gene (*PD1/PDCD1*) and programmed cell death ligand 1 (*PD-L1/CD274/B7-H1*) since they are also very prominent in the immune response of MIBC, and represent therapeutic targets for MIBC [16,17,18]. *CXCL9* (*SCYB9/MIG*) and *CXCL10* (*SCYB10*) genes are located in chromosome band 4 q21 [19], and belong to the CXC family of chemokines [20]. *CXCL9* encodes a T-cell chemoattractant that is significantly induced by interferon gamma, which mediates a T-cell-driven antitumoral immune response [21]. *CXCL9* has not been previously studied in NMIBC. The *PD1* gene has been mapped to the chromosome region 2 q37.3 by the Honyo group [22]. It encodes a cell surface receptor on T-cells and tumor-associated macrophages (TAMs), and is a member of the B7 superfamily involved in immunomodulation. PD1 acts as an inhibitory molecule on T-cells/TAMs after interacting with its ligand PD-L1 [23,24]. The *PD-L1* gene is located on chromosome 9 p24.1 and codes for a costimulatory molecule that negatively regulates cell-mediated immune responses [23,25]. PD-L1 is expressed by both tumor cells and tumor-associated antigen-presenting cells [26]. Le Goux et al. [27] did not find an association between *PD1* or *PD-L1* gene expression and prognosis (RFS and progression-free survival) in NMIBC. We recently demonstrated in an NMIBC cohort that increased *PD-L1* mRNA was an independent prognostic indicator for both RFS and DSS [28]. However, in that study, *PD1* mRNA was not associated with prognosis [28].

In this study, we analyzed a new independent cohort of NMIBC patients with extended follow-up periods to reassess the long-term association of *PD-L1* mRNA with disease prognosis, and to determine whether the two immune markers *CXCL9* and *PD1* are associated with survival.

## 2. Results

### 2.1. Correlations of CXCL9, PD1, PD-L1, KRT5 and KRT20 mRNA with Each Other and with Clinicopathological Parameters

*CXCL9* mRNA negatively correlated with the incidence of recurrence (correlation coefficient; r_s_ = −0.374; *p* = 0.001) and with mRNA of *KRT20* (r_s_ = −0.305; *p* = 0.006) and *KRT5* (r_s_ = −0.230; *p* = 0.040), and is positively correlated with mRNA of *PD1* (r_s_ = 0.639; *p* < 0.001) and *PD-L1* (r_s_ = 0.601; *p* < 0.001) (Table 1). *PD1* mRNA was negatively correlated with mRNA of *KRT20* (r_s_ = −0.253; *p* = 0.024) and *KI67* (r_s_ = −0.222; *p* = 0.047), and positively correlated with time of RFS (r_s_ = 0.298; *p* = 0.007) and *PD-L1* mRNA (r_s_ = 0.459; *p* < 0.001). *PD-L1* mRNA negatively correlated with *KRT20* (r_s_ = −0.233; *p* = 0.038) (Table 1).

### 2.2. Association of CXCL9, PD1, PD-L1, KRT5 and KRT20 mRNA with NMIBC Prognosis

The association of mRNA in the 80 tumor samples with patient survival was examined by Kaplan–Meier analysis. As expected, age was associated with both OS and DSS (*p* = 0.019 and *p* = 0.025). However, *CXCL9*, *PD1* and *PD-L1* mRNA was not associated with OS or DSS (Table 2).

**Figure 1:**
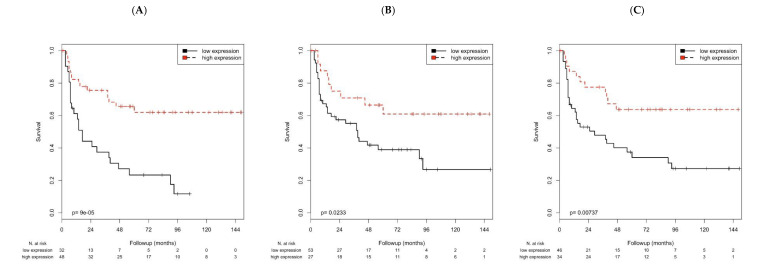
Kaplan–Meier analysis of the association of *CXCL9*, *PD1* or *PD-L1* mRNA with RFS. Gene expression was significantly associated with RFS for the genes. (**A**): *CXCL9* (*p* < 0.001). (**B**): *PD1* (*p* = 0.023). (**C**): *PD-L1* (*p* = 0.007).

Interestingly, higher *CXCL9* (*p* < 0.001), *PD1* (*p* = 0.023) or *PD-L1* (*p* = 0.007) mRNA were associated with increased RFS (all Kaplan–Meier analyses, Table 2; Figure 1).

In univariate Cox’s regression analysis, the clinicopathological parameters of histological grade, tumor stage (pT1 with/without presence of cis), intravesical therapy and gender, and the molecular parameters *KI67*, *KRT5* and *KRT20*, were not associated with prognosis (OS, DSS, RFS), and therefore were not included in further multivariate Cox’s regression analysis (data not shown).

As expected, in univariate Cox’s regression analysis, higher age (RR = 2.29; *p* = 0.022) was associated with an increased risk of shorter OS. Furthermore, higher age (RR = 3.44; *p* = 0.034) was associated with increased risk of shorter DSS (Table 3).

In univariate Cox’s regression analysis, lower *CXCL9* (RR = 3.30; *p* < 0.001), lower *PD1* (RR = 2.31; *p* = 0.027) and lower *PD-L1* (RR = 2.51; *p* = 0.009) mRNA showed an increased risk for shorter RFS. However, age was not associated with an increased risk of shorter RFS (Table 3).

In multivariate Cox’s regression analysis (adjusted for age and the molecular parameters *PD1*, *PD-L1* and *CXCL9*), an association with OS was found for higher age (RR = 2.31; *p* = 0.021) and lower *CXCL9* (RR = 2.08; *p* = 0.049) mRNA (Table 4). Multivariate analysis (adjusted for age and the molecular parameters *PD1*, *PD-L1* and *CXCL9*) revealed associations with DSS for higher age (RR = 4.47; *p* = 0.014), lower *CXCL9* (RR = 4.49; *p* = 0.006) and lower *PD-L1* (RR = 5.02; *p* = 0.042) mRNA (Table 4).

Furthermore, in the multivariate Cox’s regression analysis, associations with shorter RFS were found for lower *CXCL9* (RR = 2.69; *p* = 0.005) and lower *PD-L1* (RR = 2.07; *p* = 0.044) mRNA (Table 4).

Altogether, as expected, higher age was an independent prognostic factor for OS and DSS, but not for RFS. *CXCL9* mRNA was as independent prognostic parameter for OS, DSS and RFS. In addition, *PD-L1* mRNA was an independent prognostic factor for DSS and RFS.

### 2.3. Association of CXCL9, PD1, PD-L1, KRT5 and KRT20 mRNA with RFS Stratified by Clinicopathological Parameters or mRNA

#### 2.3.1. Stratification by Age

Using the median age of 71 years as a cut-off to define the two age groups (≤71 vs. >71 years), age itself was not associated with RFS (Table 4). In the univariate Cox’s regression analysis in the younger age group, low *CXCL9* (RR = 6.21; *p* = < 0.001) was associated with an increased risk of recurrence (Table 5). This finding is in accordance with the above mentioned results for all patients, but it indicates the greater relevance of *CXCL9* mRNA in younger patients. Low *PD1* mRNA was only associated with a risk of shorter RFS in the younger patient group (RR = 4.93; *p* = 0.035). Altogether, the higher risks of recurrence for *CXCL9* and low *PD1* levels were only relevant to the younger age group (Table 5).

#### 2.3.2. Stratification by *KRT5* or *KRT20* Expression

*KRT5* or *KRT20* mRNA is considered a characteristic feature for a basal or luminal lineage, respectively, in bladder cancer [11]. We utilized the expressions of both mRNA markers as proxies to define a more basal or more luminal-like gene expression pattern, respectively. The expression of both markers was separated by median expression into two groups with low/high *KRT5* (≤36.78 vs. >36.78) or low/high *KRT20* (≤37.47 vs. >37.47) mRNA level. In low and high *KRT20* groups, *CXCL9* mRNA was associated with a shorter RFS (RR = 3.04; *p* = 0.019 and RR = 3.28, respectively; *p* = 0.007) (Table 5). Similarly, low *CXCL9* mRNA was associated with a shorter RFS in the low and high *KRT5* groups (RR = 3.76; *p* = 0.004 and RR = 3.33; *p* = 0.013, respectively; Table 5). These results were expected since they reflected findings for all patients. In the high *KRT5* and high *KRT20* groups, low *PD-L1* mRNA was associated with shorter RFS (RR = 3.68; *p* = 0.012 and RR = 4.23, respectively; *p* = 0.009; Table 5), but this was not so in the low *KRT5* or low *KRT20* group.

#### 2.3.3. Stratification by *KI67*

*KI67* characterizes the proliferation activity of tumor cells [29]. *KI67* expression was separated into two groups (low vs. high expression) by median mRNA (≤33.10 vs. >33.10). In the high *KI67* expression group, low *CXCL9* (RR = 4.54; *p* < 0.001) mRNA and low *PD-L1* (RR = 7.49; *p* = 0.001; Table 5) mRNA were associated with a higher risk of shorter RFS, but these associations were not observed in the low *KI67* group.

#### 2.3.4. Stratification by Intravesical Therapy

Intravesical therapy was not associated with RFS in this study group. In the group with no intravesical therapy, low *CXCL9* (RR = 10.33; *p* < 0.001), low *PD1* (RR = 5.31; *p* = 0.010) and low *PD-L1* (RR = 4.36; *p* = 0.022; Table 5) mRNA was associated with the increased risk of shorter RFS, but no associations were observed with RFS in the intravesical group.

Altogether, *CXCL9* mRNA was associated with RFS in all stratification approaches. Interestingly, the increased risk of shorter RFS in low *CXCL9* mRNA patients was substantiated in the young patient group, the high *KI67* group and in patients without instillation, but it showed no association with RFS in the older patient group, the low *KI67* group or the instillation group.

In addition, the increased risk observed with low *PD1* levels was assigned to the younger patient group and the no instillation group, with no association with RFS being observed in the older patient group or the instillation patient group.

For the third marker, *PD-L1*, an increased risk of shorter RFS with low *PD-L1* mRNA was detected only in the high *KRT5* and high *KRT20* groups, but not in the low *KRT5* or low *KRT20* groups. In addition, this risk was found in the high *KI67* and the no instillation group, but not in the low *KI67* group or the instillation group.

## 3. Discussion

In this study, we investigated the mRNA of the immune markers CXCL9, PD1 and PD-L1. First, we correlated mRNA data with clinicopathological data and with each other. We observed that *CXCL9* mRNA was positively correlated with transcript levels of *PD1* and *PD-L1*, but negatively correlated with incidence of recurrence, as well as *KRT5* and *KRT20* mRNA. In addition, PD1 was positively correlated with *PD-L1* mRNA and time to RFS, while being negatively correlated with *KRT20* mRNA. *PD-L1* mRNA was additionally negatively correlated with *KRT20* mRNA.

Similar to Huang et al. we showed a correlation between the mRNA of *PD-L1* and *C-C chemokines* (*CCL2, CCL3, CCL8* and *CCL18*) [30,31]. A correlation between *PD1* and *PD-L1* mRNA was previously shown by both Huang et al. [31] and by us [28]. These correlations can all be explained by the common expression of these factors by immune cells, i.e., leukocytes such as T-cells and macrophages.

In this study, multivariate Cox’s regression analyses revealed that high *CXCL9* mRNA was associated with longer OS and DSS, and high *PD-L1* mRNA was correlated with longer DSS. In addition, the high mRNA of *CXCL9* or *PD-L1* was significantly associated with longer RFS. Huang and colleagues found that elevated *PD-L1* mRNA was associated with reduced patient survival (OS, DSS), but they studied a mixed cohort of NMIBC and MIBC where the association could have been influenced by MIBC patients, and further, they did not examine RFS [31]. We previously found that increased *PD-L1* mRNA expression was associated with longer DSS and RFS in pT1 NMIBC [28]. In this study, we confirmed the association of high *PD-L1* mRNA with DSS and RFS. However, the impact of *PD-L1* on OS, DSS and RFS need to be evaluated further in prospective studies.

*PD1* was previously not described to be associated with RFS [28], but in this study, we observed an association between increased *PD1* mRNA and longer RFS. Although both studies were performed in consecutive patients, in this study, observation time was longer (62 vs. 42 months), and the numbers of recurrences (51.3% vs. 33.4%) were higher than in the previous study, which may explain the differential results.

*CXCL9* mRNA level has not been previously described in NMIBC to be associated with OS, DSS or RFS. The effect of an immune intravesical therapy with bacillus Calmette-Guérin (BCG) on *CXCL9* mRNA was controversially discussed. BCG therapy upregulates the mRNA of different chemokines, including *CXCL9*, in an in vivo mouse model [32]. Interestingly, using an in vitro approach in established human BCa cell lines, Özcan et al. demonstrated that BCG treatment reduced *CXCL9* mRNA [33]. This supports the assumption that the tumor microenvironment is responsible for the chemokine reaction following BCG therapy. A recent review reports that the CXCL9/CXCL10/CXCL11/CXCR3 axis is responsible for angiogenesis inhibition, and the activation and migration of immune cells such as cytotoxic lymphocytes and natural killer cells into the tumor microenvironment, to prevent tumor progression in BCa [34].

Next, we were interested in whether the association of *CXCL9, PD1* and *PD-L1* mRNA with RFS could be further stratified by clinicopathological parameter (age) or other parameters applied for lineage differentiation, such as *KRT5* or *KRT20* mRNA, proliferation activity (*KI67*), or therapeutic application (instillation therapy). Interestingly, after separating patients by their median age (≤71 vs. >71 years), only in the younger age group (≤71 years) was higher *CXCL9* or higher *PD1* mRNA associated with longer RFS. This finding could be simply related to the fact that the immune system is more active in younger than in older persons, in whom immunosenescence has been reported [35]. Increasing multi morbidity affecting health status in elderly patients may also play a role in shorter RFS, although time to recurrence was not significantly different between the age groups (data not shown).

*KRT5* and *KRT20* are considered intrinsic markers for basal and luminal subtypes of muscle-invasive bladder cancer, respectively [11,36,37]. Interestingly, high *PD-L1* mRNA was associated with longer RFS in both high *KRT5* and high *KRT20* groups, but not in the low *KRT5* or low *KRT20* groups. This finding suggests that high *PD-L1* mRNA is favorable for longer RFS in both basal and luminal subtypes of NMIBC. We previously showed that high *KRT20* mRNA was associated with shorter RFS [38]. In this context, *PD-L1* mRNA further distinguishes the unfavorable RFS group (high *KRT20*) in patients with longer RFS (*PD-L1* high) or shorter RFS (*PD-L1* low).

High KI67 expression has been described as a prognostic factor for poor OS, DSS, RFS and PFS in a meta-analysis of NMIBC patients [12]. In the high *KI67* group, high *CXCL9* and high *PD-L1* mRNA were associated with longer RFS, but this association was not observed in the low *KI67* group. In this way, within the unfavorable high *KI67* group, patients with longer RFS (high *CXCL9* or high *PD-L1*) and with shorter RFS (low *CXCL9* or low *PD-L1*) could be distinguished.

Intravesical therapy with either BCG or cytostatic drugs, like mitomycin, is mostly standard therapy for intermediate or high risk NMIBC, but its application differs between several guidelines [3,5]. Interestingly, only in the no instillation group was high *CXCL9*, high *PD1* or high *PD-L1* associated with longer RFS compared to the instillation group. One explanation for this finding could be that BCG therapy affects the immune response of patients, and *CXCL9, PD1* and *PD-L1* reflect intrinsic immune status. In this way, both the expression of the immune markers and the intravesical therapy may influence each other. As mentioned above, the BCG exposure of established BCa cell lines devoid of any tumor microenvironment reduced *CXCL9* mRNA in vitro [33]. Furthermore, increases in *PD-L1* protein levels, which are considered a negative prognostic marker, have been reported after BCG therapy compared to before BCG treatment [39].

## 4. Material and Methods

### 4.1. Patients and Tumor Material

In this study, we retrospectively analyzed clinical and histopathological data from 80 patients treated with TURB at the Department of Urology and Pediatric Urology of the University Hospital Erlangen between 2000 and 2015 who were initially diagnosed with stage pT1 NMIBC (Table 6). All patients received a Re-TURB within six to eight weeks after the initial TURB. All patients were treated with a bladder-preserving approach. Tissue from formalin-fixed paraffin embedded (FFPE) tumor samples from all patients was evaluated for pathological stage according to the 2010 TNM classification [40], and was graded according to the common grading systems [41,42] by two experienced uropathologists (M.E., A.H.). All specimens contained at least 20% tumor cells. All procedures were performed in accordance with the ethical standards established in the 1964 Declaration of Helsinki and its later amendments. All patients treated after 2008 provided informed consent. For samples collected prior to 2008, the Ethics Committee in Erlangen waived the need for informed individual consent. This study was approved by the Ethics Committee of the University Hospital Erlangen (No. 3755; 2008).

### 4.2. Assessment of mRNA by qRT-PCR

Tumor specimens were assessed by qRT-PCR as previously described [43]. In short, RNA was extracted from a single 10 μm curl of FFPE tissue and processed according to a commercially available bead-based extraction method (Xtract kit; Stratifyer Molecular Pathology GmbH, Cologne, Germany). RNA was eluted with 100 μL of elution buffer. DNA was digested, and RNA eluates were then stored at −80 °C until use.

The mRNA levels of *CXCL9*, *PD1*, *PD-L1*, *KRT5*, *KRT20*, *KI67* and the reference genes *Calmodulin2* (*CALM2*) and *Beta-2 microglobulin* (*B2 M*) were determined by a one-step qRT-PCR using the SuperScript III RT-qPCR system (Invitrogen, Waltham, MA, USA) and gene specific primer-probe combinations (Stratifyer). Each patient sample or control was analyzed in duplicate in an ABI Step One PCR System (ThermoFisher, Darmstadt, Germany) according to the manufacturers’ instructions. Gene expression was quantified with a modification of the method by Schmittgen and Livak by calculating 40-ΔCt, whereas ΔCt was calculated as the difference in Ct between the test gene and the mean of the reference genes [38,44].

### 4.3. Statistical Methods

Correlations between the mRNA of *CXCL9*, *PD1*, *PD-L1*, *KRT5*, *KRT20* and *KI67* and clinicopathological data were calculated using Spearman’s bivariate correlation. Optimized cut-off values for dichotomizing each marker with respect to survival were defined using Youden’s index on the receiver operating characteristic (ROC). Detailed information about the calculated optimal cut-off values, the associated area under the ROC curve and internal validation using bootstrapping are provided in Tables S1 and S2. Following standard practice in retrospective survival analysis, the common time point zero for all patients was the date of the first TURB. The associations of mRNA with recurrence-free survival (RFS), overall survival (OS) and cancer-specific survival (CSS) were determined by univariate (Kaplan–Meier analysis and Cox’s regression hazard models) and multivariate (Cox’s regression hazard models, adjusted for age and the molecular parameters PD1, PD-L1 and CXCL9) analyses. A *p*-value < 0.05 was considered statistically significant. Statistical analyses were performed with the SPSS 21.0 software package (SPSS Inc., Chicago, IL, USA) and R V3.2.1 (The R foundation for statistical computing, Vienna, Austria).

## 5. Conclusions

Altogether, we confirmed that high *PD-L1* mRNA is associated with increased DSS and RFS. Furthermore, we demonstrated for the first time that *CXCL9* mRNA is associated with a longer OS, DSS and RFS. Associations with RFS were also identified or further pinpointed to special groups, including the younger age group (*CXCL9*, *PD1*), the high *KRT5* or high *KRT20* group (*CXCL9*, *PD-L1*), the high *KI67* group (*CXCL9*, *PD-L1*) or the no instillation group (*CXCL9*, *PD-L1*).

An increased mRNA for *PD1*, *PD-L1* and *CXCL9* being associated with a better prognosis may mirror the host–tumor interaction. In this way, we suggest that the increased mRNA levels of all three genes may reflect the immune response of the host.

Our finding of associations between these immune markers and prognosis may aid in future therapeutic options and decisions.

## Figures and Tables

**Table 1:** Bivariate correlations for mRNA of *CXCL9, KRT20, KRT5, PD1, PD-L1* and *KI67* with clinicopathological parameters.

Bivariate Correlations		*KRT20*	*KRT5*	*PD1*	*PD-L1*	*KI67*	Fu_Recurr	Recurr
*CXCL9*	Correlation coefficient	−0.305	−0.230	0.639	0.601	−0.136	0.208	−0.374
	Sig. (2-sided)	**0.006**	0.040	**<0.001**	**<0.001**	0.228	0.065	**0.001**
*KRT20*	Correlation coefficient		−0.042	−0.253	−0.233	0.356	−0.152	0.116
	Sig. (2-sided)		0.714	0.024	0.038	**0.001**	0.178	0.304
*KRT5*	Correlation coefficient			−0.212	0.036	−0.070	0.039	0.067
	Sig. (2-sided)			0.059	0.753	0.537	0.733	0.557
*PD1*	Correlation coefficient				0.459	−0.222	0.298	−0.204
	Sig. (2-sided)				**<0.001**	0.047	**0.007**	0.070
*PD-L1*	Correlation coefficient					0.001	0.096	−0.215
	Sig. (2-sided)					0.994	0.397	0.055
*KI67*	Correlation coefficient						−0.152	0.138
	Sig. (2-sided)						0.177	0.222
fu_recurr	Correlation coefficient							−0.562
	Sig. (2-sided)							**<0.001**

Abbreviation: fu recur—follow-up recurrence (time until occurrence of recurrence); recur.—recurrence. Bonferroni correction results in α = 0.00714. Significance at the α level is marked in bold.

**Table 2:** Kaplan–Meier analysis of the association of age, *CXCL9*, *PD1* and *PD-L1* mRNA with prognosis.

Parameter	Kaplan–Meier Analysis								
	*n*	OS		*n*	DSS		*n*	RFS	
		Months	*p*		Months	*p*		Months	*p*
*Age*									
≤71 vs. >71 year	40 vs. 40	124.8 vs. 84.5	**0.019**	40 vs. 40	170.2 vs. 108.3	**0.025**	40 vs. 40	n.s.	n.s.
*CXCL9*									
low vs. high	32 vs. 48	n.s.	n.s.	25 vs. 55	n.s.	n.s.	32 vs. 48	38.7 vs. 87.4	**<0.001**
*PD1*									
low vs. high	40 vs. 40	n.s.	n.s.	40 vs. 40	n.s.	n.s.	53 vs. 27	62.0 vs. 99.5	**0.023**
*PD-L1*									
low vs. high	24 vs. 56	n.s.	n.s.	46 vs. 34	n.s.	n.s.	46 vs. 34	58.6 vs. 102.7	**0.007**

Significant values are in bold face. Abbreviation: n.s., not significant.

**Table 3:** Univariate Cox’s regression analysis for the association of age and *CXCL9, PD1* and *PD-L1* mRNA with prognosis.

Parameter	Univariate Cox’s Regression Analysis								
	*n*	OS		*n*	DSS		*n*	RFS	
		RR	*p*		RR	*p*		RR	*p*
*Age*									
≤71 vs. >71 year	40 vs. 40	2.29	**0.022**	40 vs. 40	3.44	**0.034**	40 vs. 40	n.s.	n.s.
*CXCL9*						n.s.			
low vs. high	32 vs. 48	n.s.	n.s.	25 vs. 55	n.s.	n.s.	21 vs. 59	3.30	**<0.001**
*PD1*									
low vs. high	40 vs. 40	n.s.	n.s.	40 vs. 40	n.s.	n.s.	53 vs. 27	2.31	**0.027**
*PD-L1*									
low vs. high	24 vs. 56	n.s.	**n.s.**	46 vs. 34	n.s.	n.s.	46 vs. 34	2.51	**0.009**

Significant values are in bold face. Abbreviation: n.s., not significant.

**Table 4:** Multivariate Cox’s regression analysis for the association of age and *CXCL9, PD1* and PD-L1 mRNA with prognosis.

Parameter	Multivariate Cox’s Regression Analysis								
	*n*	OS		*n*	DSS		*n*	RFS	
		RR	*p*		RR	*p*		RR	*p*
*Age*									
≤71 vs. >71 year	40 vs. 40	2.31	**0.021**	40 vs. 40	4.47	**0.014**	40 vs. 40	*n*.s.	*n*.s.
*CXCL9*									
low vs. high	32 vs. 48	2.08	**0.049**	25 vs. 55	4.49	**0.006**	21 vs. 59	2.69	**0.005**
*PD1*									
low vs. high	40 vs. 40	*n*.s	*n*.s	40 vs. 40	*n*.s.	*n*.s.	53 vs. 27	*n*.s.	*n*.s.
*PD-L1*									
low vs. high	24 vs. 56	*n*.s.	*n*.s.	46 vs. 34	5.02	**0.042**	46 vs. 34	2.07	**0.044**

Significant values are in bold face. Abbreviation: n.s., not significant.

**Table 5:** Univariate Cox’s regression analysis for stratification by clinicopathological or molecular parameters: the association of *CXCL9, PD1* and *PD-L1* mRNA with RFS.

Parameter by Stratification	Univariate Cox’s Regression Analysis		
	*n*	RFS	
		RR	*p*
**Strata age: young patients**	40		
*CXCL9* low vs. high	15 vs. 25	6.21	**<0.001**
*PD1* low vs. high	27 vs.13	4.93	**0.035**
**Strata KRT5 low**	40		
*CXCL9* low vs. high	13 vs. 27	3.76	**0.004**
**Strata KRT5 high**	40		
*CXCL9* low vs. high	19 vs. 21	3.33	**0.013**
*PD-L1* low vs. high	22 vs. 18	3.68	**0.012**
**Strata KRT20 low**	40		
*CXCL9* low vs. high	13 vs. 27	3.04	**0.019**
**Strata KRT20 high**	40		
*CXCL9* low vs. high	19 vs. 21	3.28	**0.007**
*PD-L1* low vs. high	25 vs. 15	4.23	**0.009**
**Strata *KI67* high**	40		
*CXCL9* low vs. high	19 vs. 21	4.54	**<0.001**
*PD-L1* low vs. high	25 vs. 15	7.49	**0.001**
**Strata: no intravesical**	39		
*CXCL9* low vs. high	15 vs. 24	10.33	**<0.001**
*PD1* low vs. high	23 vs. 16	5.31	**0.010**
*PD-L1* low vs. high	22 vs. 17	4.36	**0.022**

Significant values are in bold face.

**Table 6:** Clinicopathological and survival data.

Clinicopathological and Survival Parameters	Patients (Percentage)
Total	80
*Gender*	
female	19 (23.7)
male	61 (76.3)
*Age (years)*	
range	46.0–97.0
mean	70.5
median	71.5
*Tumor Stage*	
pT1	52 (65.0)
pT1 with cis	28 (35.0)
*Tumor Grade 1973*	
G1	3 (3.7)
G2	28 (35.0)
G3	48 (60.0)
unknown	1 (1.3)
*Tumor Grade 2004*	
low grade	3 (3.7)
high grade	76 (95.0)
unknown	1 (1.3)
*Intravesical Therapy*	
yes	41 (51.3)
no	39 (48.7)
*Survival/observation Time (months)*	
range	0–189.0
mean	71.6
median	62.0
*Overall Survival (OS)*	
alive	44 (55.0)
dead	36 (45.0)
*Disease-Specific Survival (DSS)*	
alive	64 (80.0)
dead	16 (20.0)
*Recurrence-Free Survival Time (months)*	
range	0–149
mean	46.7
median	38.5
*Recurrence-Free Survival (RFS)*	
without recurrence	39 (48.7)
with recurrence	41 (51.3)

## Supplementary Materials

The following are available online at https://www.mdpi.com/2072-6694/12/10/2794/s1. Table S1: Optimized Ct cutoff values and internal validation and Table S2: Area under the ROC curve and internal validation.

## Abbreviations

BCa	bladder cancer
CXCL9	Chemokine, CXC motif, ligand 9
DSS	disease-free survival
Fu recur	follow up recurrence
KI67	Proliferation marker KI67
KRT5	Cytokeratin 5
KRT20	Cytokeratin 20
MIBC	muscle invasive bladder cancer
NMIBC	non-muscle invasive bladder cancer
OS	overall survival
n.s.	not significant
n.d.	not determined
PD1	programmed cell death 1
PD-L1	programmed cell death ligand 1
PFS	progression-free survival
pT	pathological tumor stage
pN	pathological lymph node stage
qRT-PCR	quantitative real-time PCR
RFS	recurrence-free survival
