# Supplementary material for: Analysis of CXCL9, PD1 and PD-L1 mRNA in Stage T1 Non-Muscle Invasive Bladder Cancer and Their Association with Prognosis
Source: Cancers (Basel). 2020 Sep 29;12(10):2794. doi: 10.3390/cancers12102794 (PMC7601021; doi:10.3390/cancers12102794)
Supplement: Supplementary file 1 [file cancers-12-02794-s001.pdf]

Supplementary Materials

Analysis of CXCL9, PD1 and PD-L1 mRNA in Stage T1 Non-Muscle Invasive Bladder Cancer and Their Association with Prognosis

Jennifer Kubon, Danijel Sikic, Markus Eckstein, Veronika Weyerer, Robert Stöhr, Angela Neumann, Bastian Keck, Bernd Wullich, Arndt Hartmann, Ralph M. Wirtz, Helge Taubert and Sven Wach

Table S1. Optimized Ct cut-off values and internal validation.

Marker	Overall Survival			Disease-Specific Survival			Recurrence-Free Survival		
	t0	Bias	Bootstrap Confidence Interval (95% Percentile)	t0	Bias	Bootstrap Confidence Interval (95% Percentile)	t0	Bias	Bootstrap Confidence Interval (95% Percentile)
CXCL9	31.92568	0.2905754	(29.62–35.53)	31.41166	0.5681872	(30.01–34.88)	31.92568	0.0455229	(31.15–33.61)
PD1	29.64531	0.05108936	(28.00–31.46)	29.99134	−0.2445304	(27.48–31.43)	30.59811	−0.6247415	(27.58–31.39)
PD-L1	29.76633	0.5161093	(29.50–31.49)	29.76633	0.4805675	(29.54–31.44)	30.69392	−0.095352	(29.69–31.05)

t0: Optimal Ct-value cutoff determined by using Youden's index; Bias estimation and confidence interval calculation based on 10,000 bootstrap replicates.

Table S2. Area under the ROC curve and internal validation.

Marker	Overall Survival			Disease-Specific Survival			Recurrence-Free Survival		
	t0	Bias	Bootstrap Confidence Interval (95% Percentile)	t0	Bias	Bootstrap Confidence Interval (95% Percentile)	t0	Bias	Bootstrap Confidence Interval (95% Percentile)
CXCL9	0.5271465	0.02333004	(0.4608–0.6562)	0.5795898	0.00443611	(0.4397–0.7351)	0.7160725	0.00057573	(0.5985–0.8251)
PD1	0.5037879	0.03439358	(0.4400–0.6480)	0.5639648	0.00949473	(0.4455–0.7168)	0.6175735	−0.00792720	(0.4335–0.7386)
PD-L1	0.5694444	0.00380602	(0.4652–0.6932)	0.5439453	0.01658122	(0.4481–0.6908)	0.6241401	0.00020403	(0.4962–0.7445)

t0: Area unter the ROC curve; Bias estimation and confidence interval calculation based on 10,000 bootstrap replicates.

© 2020 by the authors. Licensee MDPI, Basel, Switzerland. This article is an open access article distributed under the terms and conditions of the Creative Commons Attribution (CC BY) license (http://creativecommons.org/licenses/by/4.0/).
